# Dosimetric Validation of Digital Megavolt Imager for Flattening Filter Free Beams in the Pre-Treatment Quality Assurance of Stereotactic Body Radiation Therapy for Liver Metastases

**DOI:** 10.31557/APJCP.2020.21.6.1659

**Published:** 2020-06

**Authors:** Vendhan Subramani, Murali Rathakrishnan, Arunai Nambiraj N, Saraswathi Chitra S, Murali Venkatraman

**Affiliations:** 1 *Department of Radiation Oncology, Apollo Cancer Institute, Chennai, Tamilnadu, India. *; 2 *Department of Physics, School of Advanced Sciences, VIT University, Vellore, Tamilnadu, India. *; 3 *Centre for Biomaterials, Cellular and Molecular Theranostics, VIT University, Vellore, Tamilnadu, India. *

**Keywords:** SBRT QA, liver metastates, FFF beams, DMI detector, portal dosimetry

## Abstract

**Aim:**

The aim is to evaluate the use of digital megavolt imager (DMI) aS1200 in portal dosimetry with flattening filter free (FFF) beams.

**Materials and Methods:**

Dosimetric properties of DMI is characterized at 6MV FFF beams for signal saturation, dose linearity, dependency on dose-rate and source-detector distance (SDD), signal lag (ghosting), and back scatter. Portal dosimetry is done for twenty volumetric modulated arc therapy (VMAT) based stereotactic body radiotherapy (SBRT) plans for the treatment of liver metastases and the results are compared with repeated measurements of Octavius 4D.

**Results:**

The detector signal to monitor unit (MU) ratio drops drastically below 25MU. The detector linearity with dose is within 1% and no evidence of signal saturation as such. The aS1200 response variation across various dose rates and SDD is <0.4% and <0.2% respectively. The effect of ghosting increased distinctly at higher dose rate but however it is negligible (0.1%). The impact of back scatter is <0.3% because of additional shielding provided at the back of the detector. The portal dosimetry results of SBRT QA plans evaluated at the gamma criteria of 2mm/2% (DTA/DD) both under global and local mode analysis has shown an average gamma passing rate of area gamma (<1) 97.9±0.8% and 96.4±0.9%. The SBRT QA results observed in aS1200 are inline and consistent with Octavius 4D measured results.

**Conclusion:**

The characteristics of aS1200 evaluated at FFF beams have shown its potential ability as QA tool and can be used in SBRT QA for liver metastases with greater confidence.

## Introduction

Stereotactic Body Radiation Therapy (SBRT) has proved its efficacy in the treatment management of primary and metastatic liver tumors and leads to local control rates higher than 70% - 80% resulting in better survival and quality of life (Timmerman et al., 2007; Schefter et al., 2005; Scorsetti et al., 2014). The use of Volumetric Modulated Arc Therapy (VMAT) in SBRT treatments of abdominal region has definite dosimetric advantages like improved target coverage, less dose to organs at risk (OAR), healthy tissue sparing, reduced beam on time and lower number of monitor units (MU) compared to intensity modulated radiation therapy (IMRT) (Bignardi et al., 2009; Scorsetti et al., 2011).

Recent years, there has been a lot of interest in using flattening filter-free (FFF) beams in VMAT for SBRT liver metastases as it delivers the dose faster than flattened beams because of increased dose rate which leads to a significant reduction in treatment delivery time with benefit in both patient discomfort and potential limitation of intra-fraction motion (Pietro et al., 2012). In addition the removal of flattening filter was also shown to reduce out-of-field dose due to the reduction of head scatter and residual electron contamination which directly reduces radiation induced Secondary Cancer Risk (SCR) in healthy irradiated tissues surrounding the tumor (Louise et al., 2015). One of the major features of SBRT that differs from conventional radiation treatment is the delivery of large doses in a few fractions which results in a high biological effective dose (BED). The practice of VMAT based frameless SBRT with FFF beams therefore requires a high level of confidence in the accuracy of the entire treatment delivery process which including pre-treatment patient specific quality assurance.

Different methods are used for patient specific quality assurance (QA), including the use of films, ion chamber measurements and detector arrays (Ju et al., 2010; Poppe et al., 2006). Portal Dosimetry (PD) with Electronic portal imaging devices (EPID) is a most convenient tool for rapid and reliable pre-treatment verification of treatment plans. EPIDs were originally designed for patient positioning and setup verification just before the start of the treatment in radiotherapy (Van Herk et al., 1988). However soon after its implementation it was realized that the acquired real time portal images contains dose information also and could be analyzed much faster than films which led to its widespread adoption and application to perform routine quality assurance (QA) of linear accelerators and dose delivery verification of intensity modulated treatment (Kirby et al., 1995; Essers et al., 1995; Curtin-Savard et al., 1997; Antonuk et al., 1998). Amongst all the EPID systems available, flat panel amorphous silicon (aSi) based system has gained wide prominence because of its better ‘characteristics’ like faster image acquisition, high spatial resolution, high sensitivity, compact in size, stable response over time and hence better potential for dose verification (Warkentin et al., 2003; Greer et al., 2003; Van Esch et al., 2004; Louwe et al., 2004; Budgell et al., 2005; Talamonti et al., 2006).

The present generation amorphous silicon detectors in use for portal dosimetry cannot be used for measurement with high dose rate FFF beams because of factors like high dead time, slow read out electronics and effect of signal saturation and its associated problems at standard source to detector distances (SDD) of 100cm. Few authors have investigated and proposed methods of using amorphous silicon based EPID for quality assurance (QA) verification with FFF beams at extended SDDs (Eduardo et al., 2016; Min et al., 2014; Nicolini et al., 2013). A new generation of digital megavolt imager (DMI) called aS1200 EPID (Varian Medical Systems, Palo Alto, CA, USA) been introduced recently that can be used seamlessly with high dose rate FFF beams at 100cm SDD without any saturation problems. In this study the dosimetric properties of aS1200 EPID at FFF beams is characterized initially and explored its feasibility use in the pretreatment patient specific quality assurance of VMAT based frameless SBRT treatment for liver metastases with FFF beams. Further the dosimetric performance of aS1200 EPID at FFF beams in portal dosimetry for SBRT quality assurance is compared and validated against measurement results with Octavius 4D (PTW, Freiburg, Germany) and 1000 SRS (PTW, Freiburg, Germany) ion chamber detector array in place.

## Materials and Methods

**Figure 1:**
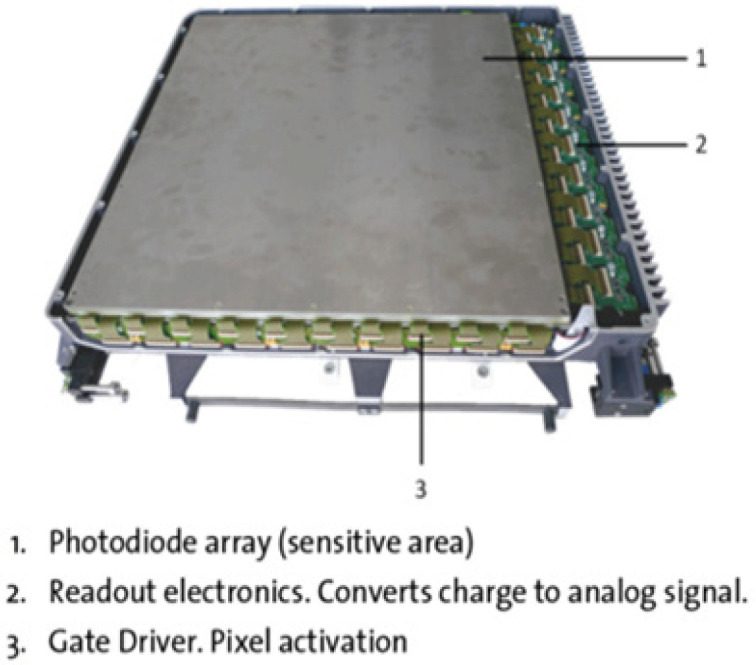
DMI (aS1200 detector)

Recently we commissioned a Varian Truebeam SVC linear accelerator (Varian Medical Systems, Palo Alto, CA, USA) at our centre. It is capable of producing 6MV FFF beam and equipped with the next generation amorphous silicon DMI detector (aS1200 EPID). The DMI detector is shown in Figure 1. The imager size of aS1200 EPID is 43x43cm2 corresponding to a pixel matrix of 1,280 x 1,280. The aS1200 EPID has higher pixel resolution (0.336mm) and increased dosimetric active area (40x40cm^2^) than its predecessor aS1000 (0.39mm and 40x30cm2). It is capable of acquiring dosimetric (acquired over all frames) images of FFF beams at high dose rates. The aS1200 has improved and advanced acquisition electronics for faster image readout. The analog to digital (A/D) conversion bit is 16 and there is no separate digitization unit to prepare images unlike in previous model EPIDs. It has a lead plate beneath the detector panel as additional shielding to reduce the arm back scatter. It can measure dose rate up to 3,200 MU/min and has an acquisition rate of 25 frames per second.

**Figure 2:**
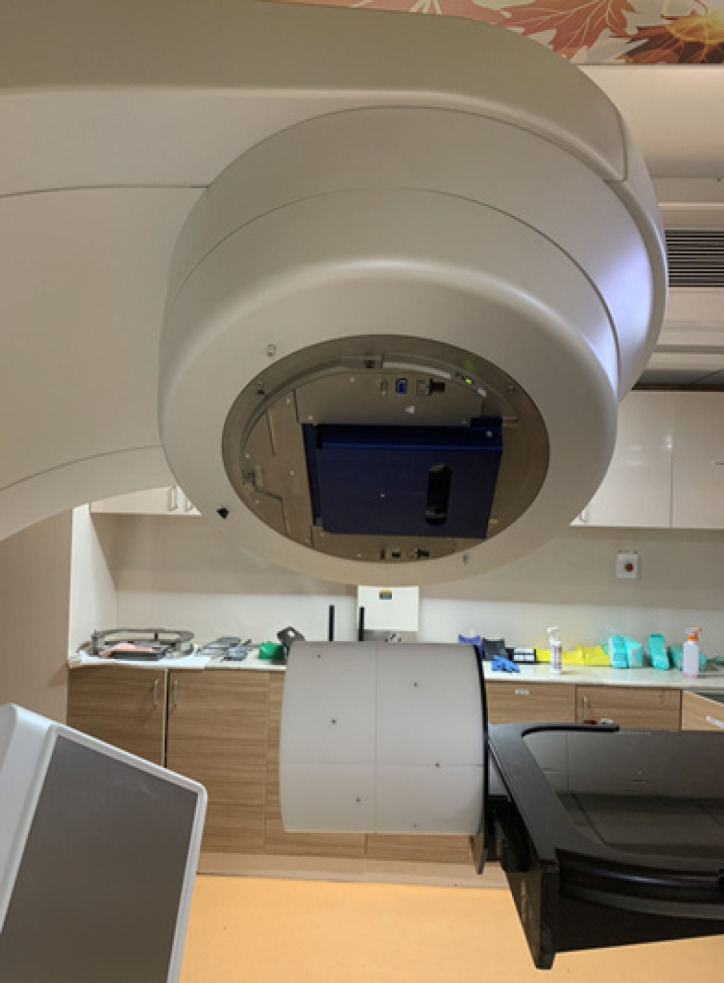
IsoCal Phantom and Collimator Plate

As a prerequisite to the study, the aS1200 EPID is calibrated for its mechanical movement, image acquisition and dosimetric measurements. In Truebeam SVC linear accelerator many of the calibration processes are auto mated. For instance, it’s enough to align the center of aS1200 EPID panel at the machine isocenter to get its arms movement auto calibrated within the mechanical limits. Dark field and flood field images are acquired automatically to calibrate the pixel response of the aS1200 imager. The system also displays the defective pixel map which gets automatically corrected. The aS1200 EPID is dose calibrated by acquiring an image at a standard source detector distance (100cm), field size (10x10cm^2^) and beam monitor units (100MU). Once the panel is dosimetrically calibrated, a relation been established between calibration unit (CU) and monitor unit (MU). Furthermore, the aS1200 EPID is geometrically calibrated using IsoCal (Varian Medical Systems, Palo Alto, CA, USA) system. The IsoCal system quickly and precisely determines the treatment isocenter of the linear accelerator and calculates image offsets for the MV imager as a function of gantry angle so that the DICOM coordinates of these MV images are exactly aligned with the treatment isocenter. The IsoCal system as shown in Figure 2 consists of a phantom, a collimator plate and application software. The phantom is a hollow cylinder 23 cm in diameter and length with 16 tungsten-carbide ball bearings (each 4 mm in diameter) located in a precisely known geometry on the surface. The collimator plate is an aluminum plate with a steel pin in its center. The plate attaches to an accessory slot in the collimator and has a spring-loaded locking system to ensure that the plate will not move with respect to the collimator upon collimator or gantry rotation. All aforesaid calibrations for aS1200 EPID are verified and monitored constantly at regular intervals and recalibrated if required. 

The first part of the study aims to validate the dosimetric response of the DMI for 6 MV FFF beam by studying its various intrinsic properties which includes signal saturation, linearity with dose, dependence on dose rate, dependence on change in source to detector distance (SDD), image lag and ghosting, and back scatter from the imager exact arm. For evaluating the linearity, the detector was positioned at 100 cm SDD and irradiated with 6 MV FFF beam at the maximum dose rate of 1400 MU/min. The dosimetric images were acquired for 3 different open square fields of 3x3cm^2^, 10x10cm^2^ and 40x40cm^2^ by irradiating with 5, 10, 15, 20, 25, 50, 75, 100, 150, 200, 250, 300, 400, 500, 750 and 1,000 monitor units (MU) respectively. To analyze the dose rate dependence, 3 different field sizes of 3x3cm^2^, 10x10cm^2^ and 40x40cm^2^ were selected and images were acquired by delivering 100 MU at various available dose rates of 400, 600, 800, 1,000, 1,200 and 1,400 MU/min.

The change in detector response with SDD was studied by acquiring images at various SDD ranging from 100cm to 150cm for 3 different field sizes 3x3cm^2^, 10x10cm^2^ cm to and 25x25cm^2^ by delivering 100 MU at the maximum dose rate of 1400MU/min. The effect of signal lag was evaluated at both minimum (400MU/min) and maximum (1,400MU/min) dose-rates by acquiring 3 images of 100MU each: a 30x30cm^2^ (reference image), next after five minutes with a 15x15cm^2^, followed immediately by irradiating second 30x30cm^2^ (ghost image) and measuring residual signal left in it. The MUs were increased to 250 and 500 for 15x15cm^2^ field and the intensity of ghosting effect was studied. Dosimetric images were acquired for 100MU at different field sizes > 8x8cm^2^ and the ratio of detector signals at ±3cm from the center pixel along the Gun-Target direction was calculated to evaluate back scatter contributions from imager support arm. Recommended setting of 2mm x 2mm Region of Interest (ROI) was used throughout the study to sample the dose in the detector.

**Figure 3:**
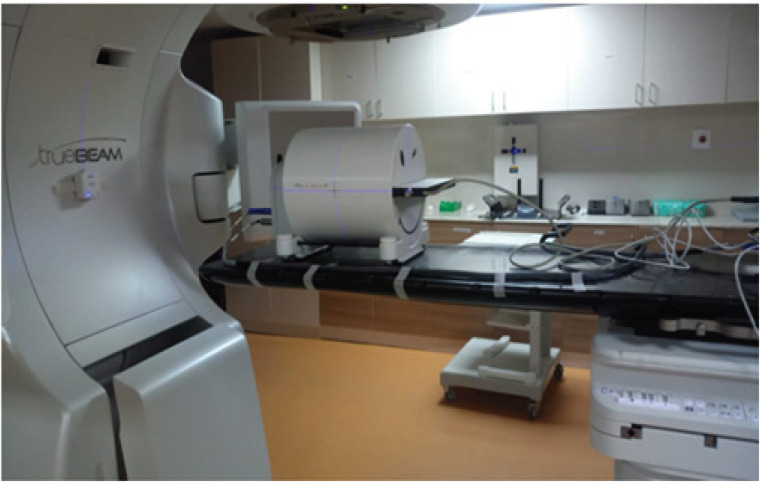
Setup of Octavius 4D Used for Measurement

In the second part of the study, the DMI feasibility is evaluated for non-transmission pre-treatment dosimetry of SBRT plans using FFF beams. For this purpose, twenty patients who already underwent frameless SBRT treatment for liver metastases in Cyberknife (Accuray Inc., Sunnyvale, CA, USA) were selected retrospectively. The treatment plans were regenerated in Eclipse TPS version 13.0 (Varian Medical Systems, Palo Alto, CA, USA) for VMAT based delivery in Truebeam SVC equipped with 120 Millennium MLC having a leaf width of 5mm at isocenter. All treatment plans were designed using two full coplanar arcs (3,600 each) employing 6MV FFF beams and a dose of 45Gy in 3 fractions was prescribed to the target volume. Pre-treatment verification plans were created using Portal Dose Image Prediction (PDIP) algorithm version 13.0 configured specifically for FFF beams. Nevertheless configuration requirements of PDIP for FFF beams were no different from flattened beams which include energy specific dosimetric calibration, output factors and kernel prediction of the imager. The EPID images were predicted at the standard SDD of 100 cm. Pre-treatment verification plans were then delivered in Truebeam SVC linear accelerator and portal images were acquired. The DMI was used to acquire portal images in the integrated mode. The measured images were analyzed and compared against the predicted images using a method of Gamma Evaluation (Low et al., 1998) by defining Distance to Agreement (DTA) and Dose Difference (DD) values in the portal dosimetry workspace in Eclipse. In this entire study, a gamma evaluation of 2 mm / 2% (DTA / DD) was used for image comparison and QA pass acceptance criteria was set as area gamma(γ <1) should be ≥ 95% of the total evaluated points. To ensure delivery accuracy of SBRT plans, all QA measurements were repeated with Octavius 4D phantom along with 1000 SRS ion chamber detector array in place. The obtained results with Octavius 4D were compared to the observed gamma results with aS1200 detector. The Octavius 4D setup used for measurement is shown in Figure 3.

**Figure 4:**
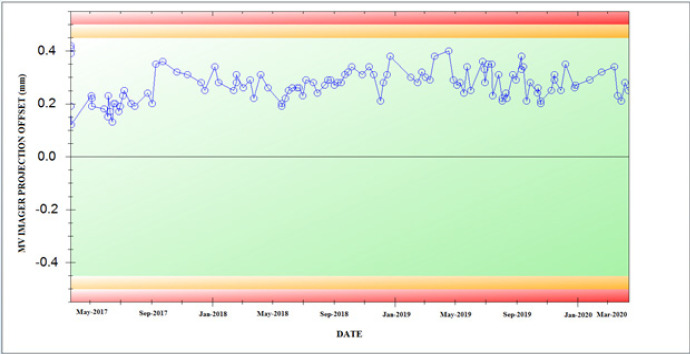
Trend of MV Imager Projection Offset

**Figure 5:**
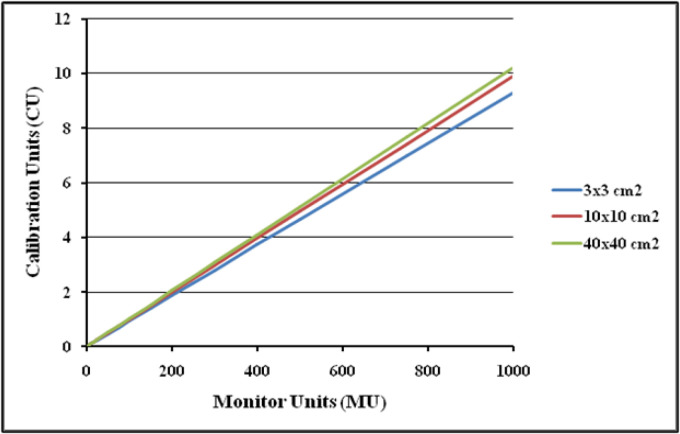
aS1200 Detector Response Linearity with Monitor Unit (or dose)

**Figure 6:**
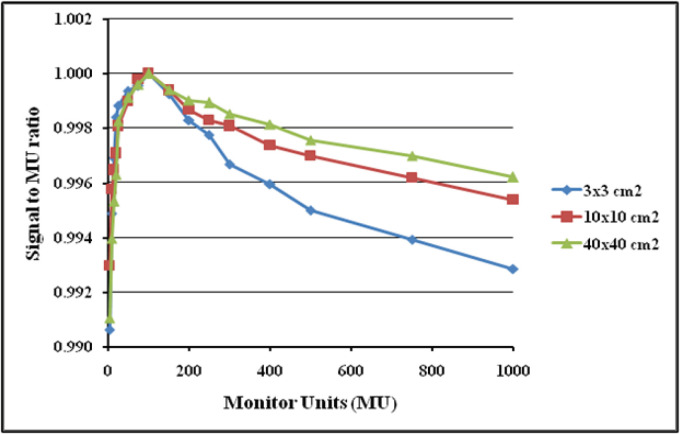
aS1200 Detector Signal to MU Ratio atDdifferent MUs

**Figure 7:**
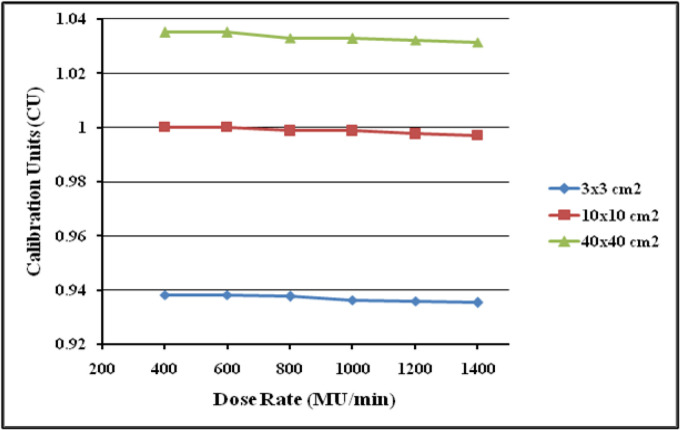
Plot of aS1200 Detector Response at Different Dose Rate (MU/min)

**Figure 8:**
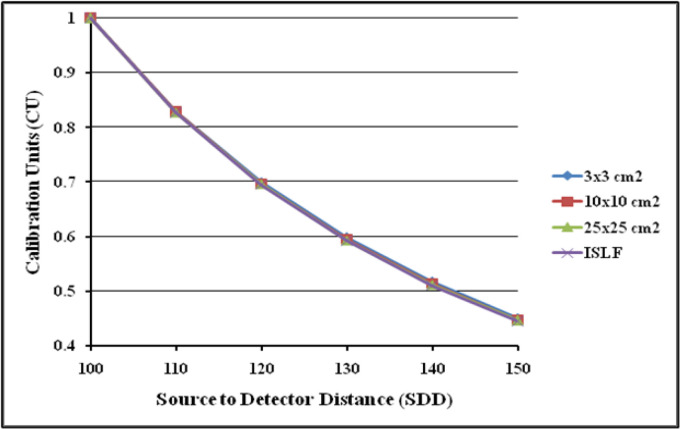
aS1200 Detector Response in Terms of Calibration Units (CU) as a Function of Source to Detector Distance

**Figure 9:**
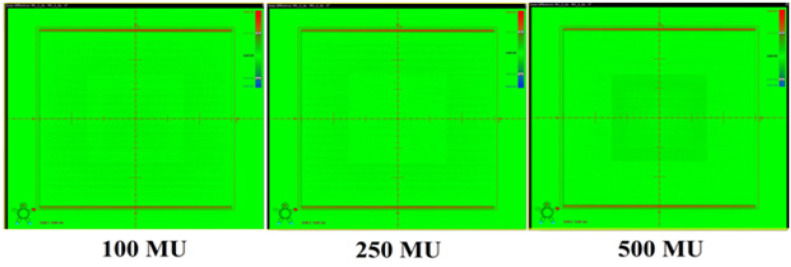
Ghosting Effect. Residual signal of aS1200 detector for different MU at low dose rate (400 MU/min)

**Figure 10:**
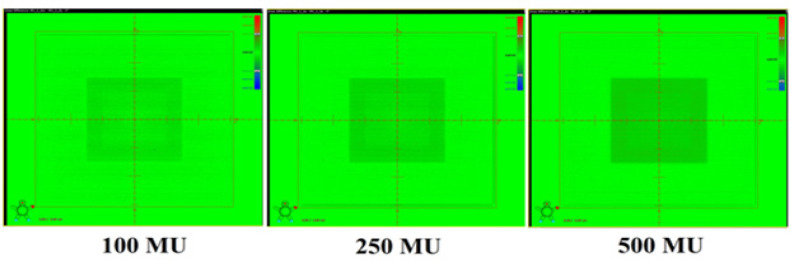
Ghosting Effect. Residual signal of aS1200 detector for different MU at high dose rate ( 1400 MU/min)

## Results

The calibration stability of the aS1200 EPID panel was continuously observed over a period of time. In the long term assessment, the MV imager panel hardly needed any recalibration with respect to both image quality and dosimetric stability as well. Figure 4 shows the trend of MV imager isocenter offset measured using IsoCal system. A mean and maximum imager isocenter offset of 0.27±0.06 mm and 0.42 mm observed respectively was a measure of the mechanical robustness of aS1200 EPID panel with gantry rotation.

The dosimetric properties of the new DMI were studied thoroughly at 6MV FFF beams. The linearity of the aS1200 detector was evaluated at a standard SDD of 100 cm. Figure 5 shows the plot of aS1200 detector response at different MUs for three field sizes. The aS1200 detector exhibits a linear dose-signal relationship as the detector signal increases linearly with increase in dose as expected. The signal to MU ratio of the detector is plotted as a function of irradiated MU as shown in Figure 6. The detector signal to MU ratio drops drastically as MU decreases especially below 25MU because of predominance of ghosting effect in detectors at lower MUs whereas for irradiations between 50MU to 1,000 MU, the variation in signal to MU ratio is 0.7%. At all field sizes, the aS1200 detector had a maximum signal to MU ratio at 100MU for unidentified reasons. The detector linearity at the dose range studied is effectively within 1% and there was no evidence of signal saturation as such. The aS1200 detector response at various dose rates of FFF beams at 100 cm SDD for three different field sizes is shown in Figure 7. The detector had a maximum variation of 0.4% in response at highest dose rate (1,400MU/min) available. From the response results it is evident that the aS1200 detector response is dose rate independent and can be seamlessly used for measurements with high dose rate FFF beams at 100cm SDD itself. The aS1200 detector response evaluated at different SDDs ranging from 100cm to 150cm in steps of 10cm had shown a maximum deviation of 0.2% at 150cm SDD for the smallest 3x3cm^2^ field size from expected inverse square law corrected value. The detector response plotted as a function of SDD at maximum dose rate of 1400MU/min is shown in Figure 8 for all three field sizes. The signal lag of the aS1200 detector at FFF beam was determined by the amount of residual signal measured after successive irradiations. Signal lag was measured both at lowest (400MU/min) and highest (1,400MU/min) dose rate available. The ghosting effect as measured is shown in Figure 9 and Figure 10 for low and high dose rate respectively. The ghosting effect was seen distinctly at high dose rate in comparison with low dose rate. As the MU irradiated on the detector increased, the effect of ghosting also increased but however it is negligible. A maximum of 0.1% increase in detector signal was observed for an irradiation of 500 MU. The effect of scatter radiation arising from the back arm holding the detector on the quality of acquired image was studied at different field sizes. A maximum signal variation of 0.3% was observed and the back scatter component from the arm had no correlation with increase in field size because of additional shielding provided at the back of the detector panel.

Portal dosimetry was carried out with aS1200 DMI detector for pre-treatment quality assurance of 20 SBRT liver metastases plans which utilizes FFF beams. Gamma analysis was used for comparing acquired portal image with predicted image. All SBRT QA plans evaluated at the gamma criteria of 2mm/2% (DTA/DD) both under global and local mode analysis shown a higher gamma passing rate (>95%) with an average area gamma (<1) of 97.9±0.8% and 96.4±0.9% of the evaluated points respectively. Only pixels with a threshold of ≥5% of the maximum dose or calibrated unit (CU) in complete irradiated area outline (CIAO) were included for comparison in the computation of gamma. The observed gamma passing rate with aS1200 detector in SBRT QA verification which paves for collective evaluation at both high dose rate (>1,000 MU/min) and high dose range (>10Gy), were clear indicative of the feasible use of aS1200 with FFF beams. 3D gamma analysis of measurements made in Octavius 4D along with 1000SRS detector for the same SBRT QA plans fetched an average passing rate of 97.1±1.1% adding conformance to the delivery accuracy of plans and together validates the portal dosimetry results with aS1200.

## Discussion

The inherent response characteristics and dosimetric properties of the new DMI (aS1200) detector at FFF beams were studied. The results suit the use of aS1200 at both high dose range (>10Gy) and high dose rate (>1000MU/min) as well. Our study on pretreatment QA verification of VMAT based SBRT in liver metastases with aS1200 had highlighted the potential ability of aS1200 as a reliable QA tool for FFF portal dosimetry.
